# A novel method to compare protein structures using local descriptors

**DOI:** 10.1186/1471-2105-12-344

**Published:** 2011-08-17

**Authors:** Paweł Daniluk, Bogdan Lesyng

**Affiliations:** 1 Faculty of Physics, Department of Biophysics and CoE BioExploratorium, University of Warsaw, Żwirki i Wigury 93, Warsaw, Poland; 2 Bioinfomatics Laboratory, Medical Research Centre, Polish Academy of Sciences, Pawińskiego 5, 02-106 Warsaw, Poland

## Abstract

**Background:**

Protein structure comparison is one of the most widely performed tasks in bioinformatics. However, currently used methods have problems with the so-called "difficult similarities", including considerable shifts and distortions of structure, sequential swaps and circular permutations. There is a demand for efficient and automated systems capable of overcoming these difficulties, which may lead to the discovery of previously unknown structural relationships.

**Results:**

We present a novel method for protein structure comparison based on the formalism of local descriptors of protein structure - DEscriptor Defined Alignment (DEDAL). Local similarities identified by pairs of similar descriptors are extended into global structural alignments. We demonstrate the method's capability by aligning structures in difficult benchmark sets: curated alignments in the SISYPHUS database, as well as SISY and RIPC sets, including non-sequential and non-rigid-body alignments. On the most difficult RIPC set of sequence alignment pairs the method achieves an accuracy of 77% (the second best method tested achieves 60% accuracy).

**Conclusions:**

DEDAL is fast enough to be used in whole proteome applications, and by lowering the threshold of detectable structure similarity it may shed additional light on molecular evolution processes. It is well suited to improving automatic classification of structure domains, helping analyze protein fold space, or to improving protein classification schemes. DEDAL is available online at http://bioexploratorium.pl/EP/DEDAL.

## Background

The methods of protein structure alignment play a crucial role in computational and structural biology. However, despite extensive research, comparison of protein structures still remains an open subject. Even in the category of the most straightforward approaches which focus on finding the largest possible sets of superimposable amino-acids, treating structures as rigid entities and preserving the order of aligned residues, there is no definitive "best of all" method [1]. Furthermore, there exists a growing set of known biologically significant similarities between protein structures with considerable spatial distortions, various segment swaps or circular permutations [2-5]. These "gold standard" alignments are prepared with substantial human intervention [6] and studies have shown that no automated techniques to date are capable of satisfactorily reproducing them [7].

The reason behind the aforementioned problems is the fact that proteins which in fact are fairly elastic objects are represented by fixed atomic coordinates in 3D space, usually obtained from crystallographic experiments and most methods focus on finding a superposition which would minimize the distance between the respective amino-acids. Such a paradigm greatly simplifies the difficult task of identifying equivalent residues and thus may be very appealing, but is incapable of distinguishing between regions which are strongly stabilized by actual protein interactions and those which are of looser composition. The major approaches to structure superposition, including comparing intramolecular inter-residue distances (SSAP [8], DALI [9], PAUL [10]), matching main-chain fragments (CE [11]), or Secondary Structure Elements (SSEs) (VAST [12], SARF [13], MATRAS [14], GANGSTA [15]), handle the limitations imposed by the rigid-body representation with varying degrees of success. Some methods use residue attached local frames of reference to identify partial superpositions which are then clustered (C*_α_*-match [16], 3D motifs [17], growing neighborhoods [18]). In principle, this approach allows for sequential rearrangements. The final alignment is inferred from the predominant superposition. Other methods use a one-dimensional representation of structure, where each residue is substituted with a characterization of its local features, and use dynamic programming to align such artificial sequences (e.g. SHEBA [19]). Still others employ alternative ways of describing protein structure, including Delaunay tessellation (TOPOFIT [20]), spherical polar Fourier representations (3D-BLAST [21]), and geometric hashing (C*_α_*-match [16]). To specifically address structural shifts and distortions, some methods search for "hinges" between superimposable rigid parts (FATCAT [22], FlexProt [23], ProtDeform [24], FlexSnap [25]). For an alternative classification, see a recent review [26].

Methods which attempt a decomposition of protein structures to smaller blocks are most likely to suffer from combinatorial complexity. While in principle they should be capable of finding alignments unconstrained by amino-acid sequence (i.e. with permutations or segment swaps), finding such an alignment is likely to be computationally prohibitive. Therefore, most approaches do not allow for sequential rearrangements. This is of less importance in the case of the methods using relatively large SSEs. However, one of the disadvantages of using SSEs is that the active sites are frequently small and contained in the coiled regions, and it is particularly important to align these correctly. Another method of curbing combinatorial complexity is to use the scoring function based on the rigid-body superposition, possibly allowing for "hinges" between superposable rigid parts. To date, we are aware of only one method capable of computing non-rigid alignments with sequential permutations (FlexSnap [25]). It should also be noted that methods tailored to the particular problem do not perform as well as mainstream approaches on the regular simple comparisons.

Finding an elegant way to address the aforementioned difficulties has been a motivation behind developing DEDAL. It is based on a formalism for representing and comparing local structure, the so called Local Descriptors of Protein Structure (LDPS) [27,28]. In a much simpler implementation (called DAL) it has been used to identify regions of correctly predicted structure in models submitted to CASP [29,30]. A single local descriptor contains information about the structure within a range of bonded and non-bonded interactions of a single amino-acid. Therefore, contrary to backbone segments or SSE, it can be treated as a complete self-contained structural entity. Alignments built from such blocks preserve contacts, which correspond to physical interactions between residues. Descriptors are large and specific enough to lessen the combinatorial burden and omit the sequential constraints. There is no thus need to use a global RMSD [31,32] or other rigid-body measure to verify the feasibility of alignments.

## Results

### Algorithm

**Figure 1:**
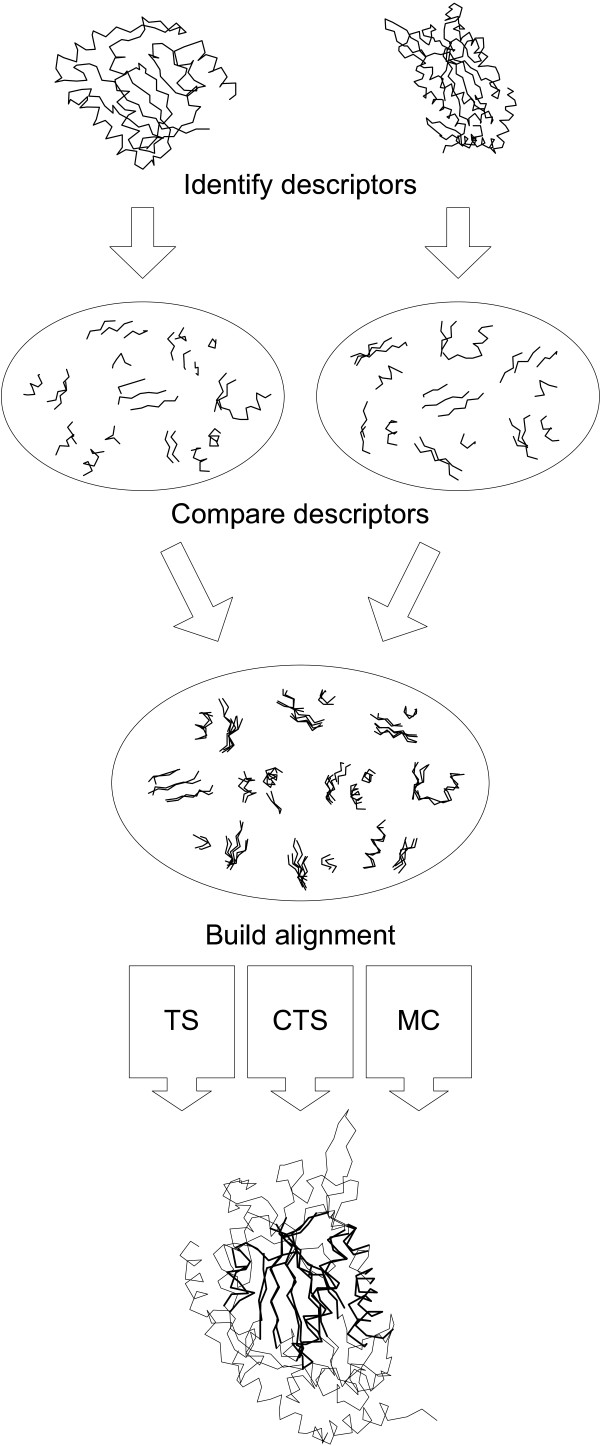
**Overview of the DEDAL algorithm**. The procedure comprises three stages: (1) identification of descriptors in compared molecules; (2) comparing them all against all to find a set of local substructures common to both molecules; and (3) building an alignment of identified local similarities using one, or a combination of TS, CTS and Monte Carlo algorithms (see text).

Our method performs comparison based on local structural similarities. After all of the local descriptors in each structure are identified, they are compared against each other. Pairs of similar descriptors are then used as building blocks for the alignment. For a schematic diagram of the alignment process see Figure 1.

**Figure 2:**
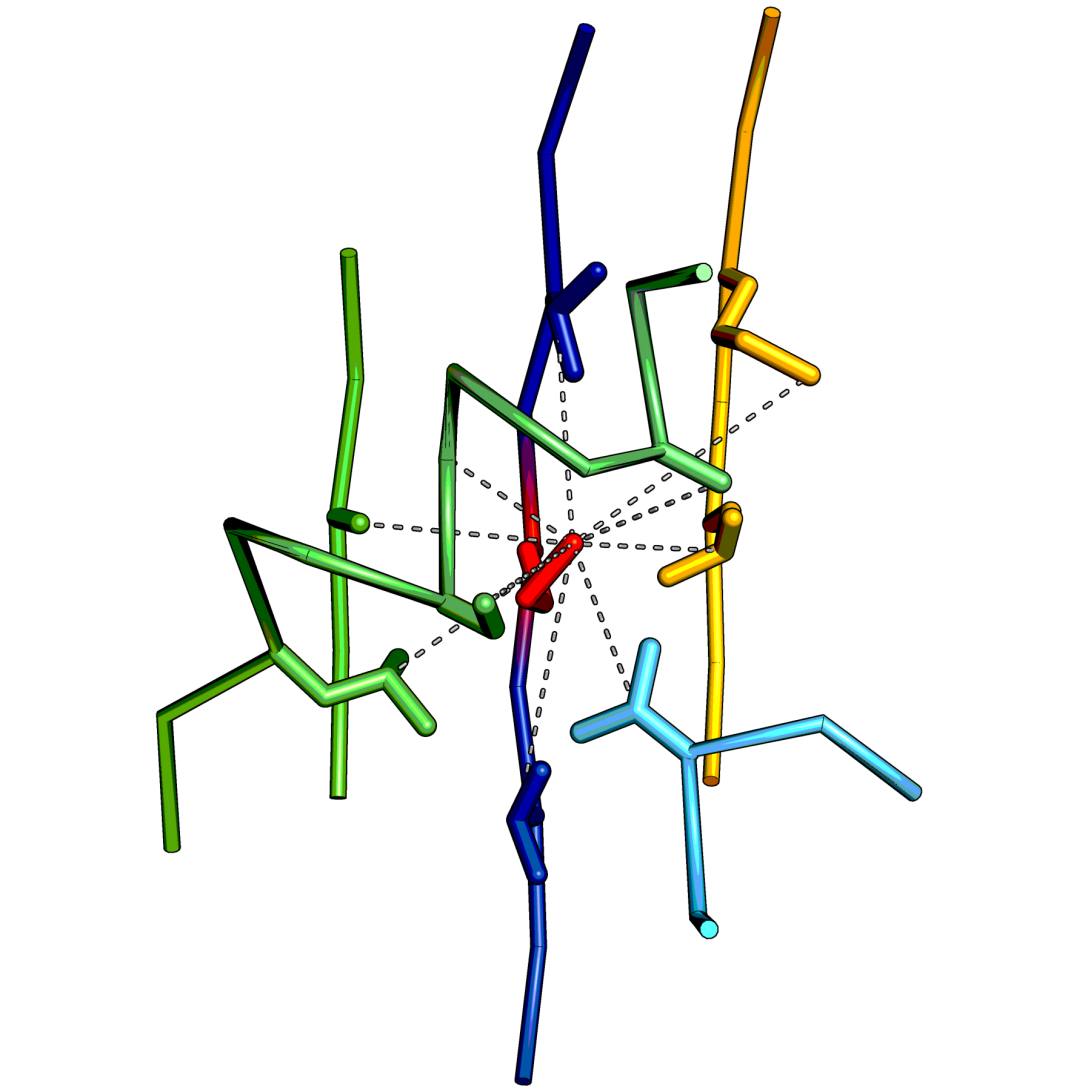
**Sample descriptor (built around the residue **MET70** of the SCOP domain **d1lg7a_**)**. This descriptor contains 9 contacts (dashed lines) between its central amino-acid (red) and residues forming the centers of its elements. Some of the elements overlap forming longer segments (in particular fragments of two *β*-strands (blue and yellow) and fragment of *α*-helix (green)). Altogether, this descriptor comprises 5 continuous segments.

A local descriptor is a small part of a structure that can be viewed as a residue-attached local environment. In principle, it is possible to build a descriptor for every residue of a given protein. This process begins by identifying all residues in contact with the descriptor's central residue. Elements are then built by including two additional residues along the main-chain, both upstream and downstream of each contact residue. Any overlapping elements are concatenated into single segments. Thus, a descriptor is typically built of several disjoint pieces of the main chain (Figure 2). It reflects approximately the range of local, most significant physico-chemical interactions between its central residue and other amino-acids. This constitutes a significant difference compared to the single segments so frequently used in other studies. Single segments reflect features along the main-chain, while descriptors are spatial, and thus add a three-dimensional context to the local properties of proteins.

Using more complex building blocks resulted in several problems which had to be solved. The first was assessing similarity between descriptors. In the case of single segments of the same length, it is easy to compute RMSD between respective amino-acids. When comparing descriptors, we first have to identify the mapping between their residues and only afterwards can we compute the RMSD between them. We consider the two descriptors similar and the resulting sequence alignment valid only when descriptors display sufficiently similar residue-residue contact patterns and the corresponding RMSD is sufficiently small. In our implementation we search for all alignments with a total RMSD not exceeding 2.5Å, and such that at least half of the segments in each of the descriptors are aligned.

To extend the alignment to whole structures we employ a three-stage process. First, we find all pairs of similar descriptors and their respective structural alignments. To discover significant similarities between local structures and avoid small accidental matches, we consider only alignments that consist of at least three segments, postponing the use of the two- and single segment descriptors to the final stage of the algorithm. Each such alignment can be considered as a building block for the alignment of whole structures. Obviously, not all blocks fit together, but those that do can be combined into larger alignments. In the second stage, we identify the largest sets (with respect to the number of residues) of non-conflicting descriptor pairs, i.e. the largest building block assemblages. From a mathematical point of view, this is a clique finding problem. In the final stage we use the remaining descriptor alignments, which were previously set aside, and add them to alignments from the second stage, but only if they overlap with the alignment being built. The resulting alignments have the following properties:

• Each pair of aligned amino-acids belongs to at least one pair of aligned descriptors, which implies that their respective local neighborhoods are preserved,

• There does not necessarily exist a superposition of aligned amino-acids, the alignment may have to be divided into several independently superposable parts,

• Alignments may contain permutations of segments.

Our approach is of a non-rigid-body type but, contrary to other non-rigid-body methods, it does not attempt to find "hinges" which might make superposition possible. Rather, it ensures that alignment can be broken into separately superimposable regions, which are large enough to be structurally meaningful. In particular, separating stages two and three guarantees that each region will contain at least one three-segmented descriptor. The third property provides the ability to handle "difficult similarities". During the process of building an alignment, no restrictions are placed on the order of segments in the resulting mapping. Therefore, two similar proteins with different threading, but similar arrangements of secondary structure elements, can still be aligned. Using the terminology employed by CATH [33], it is possible to align two structures of the same architecture, even if their topologies are different.

The algorithm can be adjusted by modifying the internal scoring function. The most basic score is simply the number of aligned residues. It is also possible to limit the maximal offset between aligned residues. If the lengths of compared chains differ then offset is measured relative to the closest of shortest possible (i.e. ungapped) alignments or allowing gaps only in the shorter of the two protein chains. Sometimes, it is undesirable to find alignments with permutations. In such cases it is possible to take the largest sub-alignment which has no more than a given number of swaps. This, for example, permits searching explicitly for circularly permuted proteins.

As mentioned above, DEDAL is not restricted to finding rigid-body superpositions. This feature can be exploited in two ways. Firstly, it can be used to discover several disjoint, differently arranged similar substructures within one pair of proteins (e.g. domains or subdomains). Secondly, it can be used to address minute local differences which in a gradual continuous way may result in a global RMSD too large to handle for the traditional rigid-body methods.

### Testing

#### Datasets

The performance of structure superposition methods is commonly tested by (a) rigid-body RMSD and (b) the extent of the obtained superposition. While in many cases this is a valid approach, in many others alignments containing local alignment errors (induced by spatial proximity of residues rather than common architectural features or local similarity of the compared structures) are indistinguishable from correct ones. This in turn may result in misleading assessments of performance, especially in cases of low structure similarity, at which DEDAL is primarily aimed. Therefore, we resort to the manually curated structural alignments and a simple measure of how accurately they are reproduced by the automated approach. The numerical measure we use is the ratio of the number of residue pairs aligned in the same way in both the computed and curated alignments, and the size of the curated alignment. As a reference we use alignments compiled in (a) the SISYPHUS database [6], (b) the SISY set, a subset of the SISYPHUS database prepared by Mayr et al. [7], and (c) the RIPC set, containing selected challenging alignments, also prepared by Mayr et al. and based on the SCOP database [34]. Using SISY and RIPC sets allows for a direct comparison with the Mayr et al. study.

The SISYPHUS database contains manually curated alignments for proteins with non-trivial relationships, which are divided into three categories (fragment, homologous sequence, fold). Similarities in the homologous sequence and fold categories are usually large enough to encompass a significant portion of the aligned structures and thus present a good benchmark for the structural alignment software. Each multi-alignment in SISYPHUS consists of at least two structures with a common substructure. It frequently occurs that some of these structures are almost identical. We chose to filter out all structures with at least 80% of residues superimposable within the distance not greater than 2Å. This was done with the LGA structure alignment program [35]. A greedy algorithm was used to prune such redundant examples leaving only one specimen for each set of similar structures. After the initial pruning, the remaining 113 multi-alignments were assigned to one or more of the three categories:

1. SCOP - alignments comprising structures which can be related to domains in the SCOP database,

2. MD - alignments containing multi-domain structures,

3. MC - alignments containing multi-chain structures.

Machine parsable lists of alignments can be found in Additional files 1, 2 and 3.

Structures in the PDB very often contain multiple chains filling a unit cell within a crystal. An undesired redundancy may be created if the entire contents of the unit cell are compared. Therefore, we have used the PDB "biological units" whenever possible.

The SISY set contains 69 non-redundant pairs selected from the SISYPHUS database by Mayr et al. [7]. From each SISYPHUS multiple structure alignment they have selected the pair with the lowest sequence identity. Pairs with more than 40% identity or those including structures comprised of multiple chains were excluded.

The RIPC set comprises 40 pairs of SCOP domains also selected by Mayr et al. [7]. These, albeit structurally related, are difficult to align due to repetitions, extensive insertions/deletions, circular permutations and/or considerable conformational variability. For 23 of these pairs, the authors provide reference alignments supported by evidence of sequence and function conservation.

#### Reconstruction of SISYPHUS alignments

We have executed the TS+CTS and CTS+CTS algorithms (see Methods) on all pairs of structures from the pruned SISYPHUS alignment set, computing for each algorithm at most the 5 largest alignments which differ significantly, and selecting the one that was most similar to the alignment curated in the SISYPHUS database. Computing more than one alignment is necessary because the SISYPHUS reference alignment is not always the largest or the best one, for example when the compared structures contain repeated motifs or internal symmetries which make alternative superpositions/alignments possible. This fact has been also noted by Mayr et al. [7].

**Figure 3:**
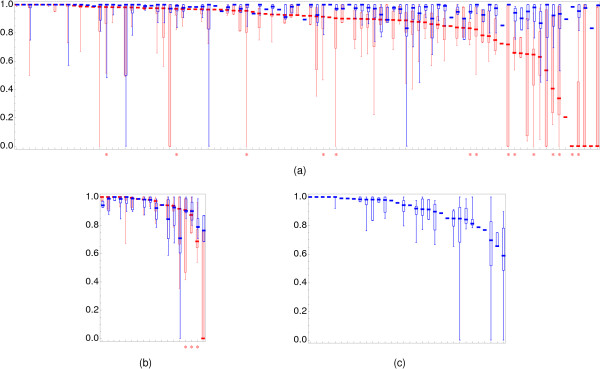
**Comparison of performance of DEDAL and DALI on SISYPHUS alignments**. The quality with which the SISYPHUS alignments are reproduced by DEDAL (blue) and DALI (DaliLite, red) on the (a) SCOP, (b) MD (multi-domain), and (c) MC (multi-chain) subsets of the dataset (see text for description), presented as box-and-whisker distribution plots of the agreement with the reference alignments. Each entry corresponds to a set of pairwise comparisons carried out for each of the SISYPHUS multi-alignments. The entries are ordered by the performance of DALI (on SCOP and MD sets), or DEDAL (on the MC set). Box-and-whisker plots represent lower and upper quartiles (box) relative to the median (horizontal line) of the results obtained for a given SISYPHUS multi-alignment, as well as minimum and maximum values (whiskers). When a distribution is reduced to the alignment of a single pair of structures the box-and-whisker plots are correspondingly reduced to a single value. Asterisks indicate alignments containing segment swaps, or circular permutations. For numerical results see Additional files 5, 6 and 7. While DALI performs slightly better than DEDAL on the easier cases (left-hand-side of the SCOP set), DEDAL does better on more difficult and multi-domain alignments (DALI does not align multi-chain proteins).

For the single chain structures we repeated this experiment using DALI [36] with the default settings. The selection of DALI for comparison purposes was based on its having the best performance in the Mayr et al. comparison [7]. If more than one alignment was returned, we again selected the one most similar to the SISYPHUS alignment. Ultimately, for each algorithm and each pair of structures in the dataset, we computed a score equal to the percentage of amino-acid pairs correctly aligned (Figure 3, and Additional File 4, Figure S1, as well as Additional files 5, 6 and 7). Both methods show similar performance in the case of easy similarities, with DALI possibly registering a slight advantage over DEDAL. However, similarities which are problematic for DALI (right hand side of the box-and-whisker plots) are solved well by DEDAL. The average performance of DEDAL on the SISYPHUS dataset is 90% (with the median of 95%). This compares to 90% for DALI (median of 97%). When comparing results of DEDAL with DALI, it should be noted that the DALI alignments are built using smaller blocks, and thus very seldom leave unaligned residues. Descriptors are larger, frequently leading to alignments which could easily be extended by a few residues, without lowering the quality of the superposition, but due to the fairly large granularity of descriptors, there are no pairs of similar descriptors which could facilitate this. It should also be noted that DEDAL performs well on multi-domain (Figure 3b) and multi-chain structures (Figure 3c), while DALI is not capable of dealing with multiple chains, and performs less well if the spatial orientation of multiple domains differs between structures. It should be noted that for MD and MC sets we report only those alignments which involve at least one multi-domain or multi-chain structure. Alignments of single domains are reported in the analysis of the SCOP set.

#### Reconstruction of the SISY and RIPC alignments

**Figure 4:**
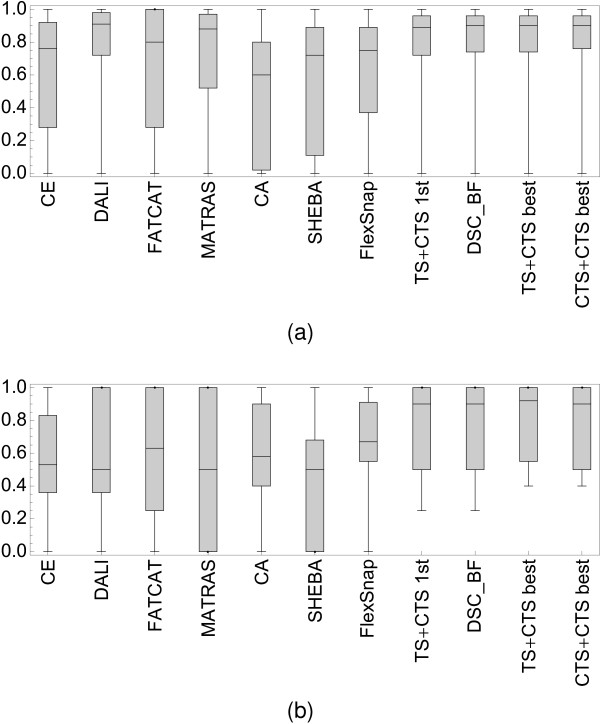
**Comparison of performance of DEDAL and other methods on the SISY and RIPC datasets**. The quality with which the reference alignments in the (a) SISY, and (b) RIPC sets (see text) are reproduced by DEDAL and other methods. Box-and-whisker distribution plots (see legend to Figure 6) of the agreement with the reference alignments are shown for each method. DEDAL results are shown for both the TS+CTS and CTS+CTS regimes, including scores for the first and best of the five calculated alignments, as well as for the best of both methods. All other results are from Mayr et al. For numerical results see Additional files 8 and 9. On the SISY set, the performance of DEDAL (76% average accuracy, 89% median accuracy) is comparable with that of DALI (76%, and 91% respectively). The third ranked MATRAS achieves 68% average accuracy (88% median). On the more challenging RIPC set, DEDAL significantly outperforms other methods (see also Tables 1 and 2 for the analysis of significance).

We have also used the protocol described for the SISYPHUS dataset above to generate alignments for the SISY and RIPC sets. We have compared the results of the TS+CTS and CTS+CTS algorithms (see Methods) with results for CE, DALI, FATCAT, MATRAS, CA and SHEBA as computed by Mayr et al. [7] and FlexSnap [25] (Figure 4a, see also Additional File 4, Figure S2, as well as Additional files 8 and 9). Mayr et al. only provide results for the first alignment computed by the methods they have tested. In the case of the DEDAL results, one very seldom observes an improvement when selecting alignments other than the first one from the set of five best computed. We provide results for both the first, and the best-of-five alignments obtained with the TS+CTS and CTS+CTS computations. For consistency, only the first alignments are used in the significance analysis. Box-and-whisker plots (Figure 4a) show that DEDAL performs at least as well as DALI and MATRAS. The mean accuracy on the SISY set is 76% (median of 89%), while DALI achieves 75% (91%), and MATRAS - 67% (88%). The difference is larger for the alignments on the RIPC set (Figure 4b), where the lower quartile of the quality for DEDAL's TS+CTS alignments is comparable to the median for other methods. DEDAL's average accuracy is 77% (median of 90%), while the second best FlexSnap achieves 66% (median of 67%). DALI has average accuracy of 60% (median of 50%). The distributions of the accuracy scores were compared using the two-sided Wilcoxon signed rank test with paired observations (Tab. 1, 2). On the SISY set DEDAL (in the TS+CTS mode) performs significantly better than CE, CA, SHEBA and FlexSnap (p-values of 1 × 10^-4 ^or lower). It also performs better then FATCAT (p-value 3.5 × 10^-2^) and MATRAS (although the difference in this case is not significant), and performs on a par with DALI. On the more difficult RIPC set, it performs significantly better than all other methods (RIPC is smaller than SISY, and therefore all p-values are larger).

**Table 1:** Results of the Wilcoxon test for alignment accuracy in the SISY set

	DALI	FATCAT	MATRAS	CA	SHEBA	FlexSnap	TS+CTS
CE	3.7·10^-5^	2.7·10^-1^	1.5·10^-2^	6.6·10^-2^	2.5·10^-1^	9.6·10^-1^	1.0·10^-4^
DALI		1.4·10^-2^	1.2·10^-2^	2.2·10^-8^	9.5·10^-7^	2.0·10^-5^	1.3·10^-1^
FATCAT			3.6·10^-1^	9.3·10^-3^	1.4·10^-1^	6.0·10^-1^	3.5·10^-2^
MATRAS				5.5·10^-5^	6.9·10^-4^	2.3·10^-2^	4.2·10^-1^
CA					9.2·10^-3^	7.5·10^-3^	4.8·10^-9^
SHEBA						4.6·10^-1^	2.0·10^-6^
FlexSnap							8.5·10^-5^

**Table 2:** Results of the Wilcoxon test for alignment accuracy in the RIPC set

	DALI	FATCAT	MATRAS	CA	SHEBA	FlexSnap	TS+CTS
CE	1.9·10^-1^	3.3·10^-1^	3.6·10^-1^	4.8·10^-1^	8.4·10^-2^	1.3·10^-1^	3.9·10^-3^
DALI		3.7·10^-1^	2.9·10^-1^	3.4·10^-1^	2.2·10^-2^	2.7·10^-1^	2.9·10^-2^
FATCAT			3.5·10^-1^	3.4·10^-1^	2.1·10^-2^	2.3·10^-1^	3.3·10^-2^
MATRAS				4.8·10^-1^	8.4·10^-2^	2.3·10^-1^	2.9·10^-2^
CA					9.8·10^-2^	4.1·10^-2^	5.9·10^-4^
SHEBA						3.2·10^-2^	1.2·10^-3^
FlexSnap							1.3·10^-2^

### Implementation

We have implemented the described algorithms in C on the Linux platform. The typical running time of a single comparison of a pair of structures using the TS and CTS algorithms ranges from seconds to a few minutes (on a 2.6 GHz AMD Opteron CPU), depending on the number of pairs of similar descriptors. In some cases, when structures are composed of several similar subdomains (e.g. propeller folds), the running time can reach several hours. We extracted 14 of the most computationally intensive cases and used them to test the REMC algorithm. We have experimentally determined the optimal number of replicas, the frequency of replica exchanges and the number of iterations required to reach globally the maximal score. The running time of the Monte-Carlo algorithm is mostly dependent on these three factors, therefore typically any pair of structures can be aligned in a few minutes. In the experiments described above, the REMC algorithm was used as a fallback option in the cases where combinatorial algorithms failed to finish in 120 seconds.

We have made DEDAL available online at http://bioexploratorium.pl/EP/DEDAL. The website also provides Linux binaries of the software. The server can be used to align structures identified by PDB or SCOP accession codes or supplied in uploaded files. Both TS+CTS and CTS+CTS algorithms are available, along with other modes potentially useful for the advanced user to cope with special cases, or to provide more insight into the behavior of DEDAL. It is also possible to define the parameters of the scoring function (*k *- maximal sequence offset, *M *- maximal number of swaps in the permutation, as explained in the Methods section). Results are presented in HTML format. Superpositions can be downloaded as PDB files or RasMol scripts, and also viewed through the Jmol applet. The alignments are available in FASTA format and as a list of corresponding residue ranges.

## Discussion

### Case studies

To illustrate the capacity of the descriptor based approach we present three cases of difficult structure alignments not handled effectively by methods limited by the rigid-body or sequence-dependence constraints.

#### Saposins

**Figure 5:**
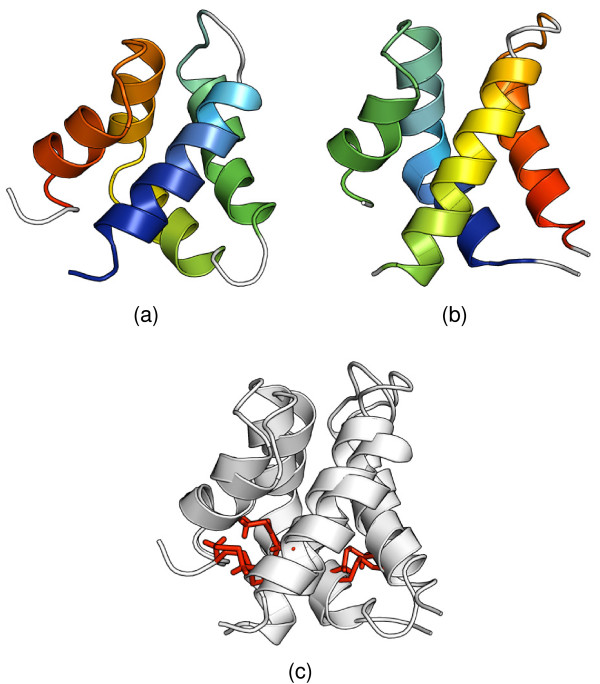
**Alignment of the Saposin domain of NK-lysin and the "swaposin" domain of prophytepsin**. (**a**) The Saposin domain of NK-lysin (SCOP domain d1nkla_) and (**b**) the "swaposin" domain of prophytepsin (d1qdma1). Despite different topology these two domains have the same architecture and identical disulfide bonds. (**c**) DEDAL correctly identifies the best superposition and the disulfide bond network (the sequence identity between these molecules is 14.5%).

The circular permutation between saposin and saposin-like "swaposin" domains is one of the very first discovered of its kind. The discovery was made by sequence analysis [37], and verified when the crystal structures became available. NK-lysin (SCOP domain d1nkla_) comprises five *α*-helices, conforming with the "folded leaf" architecture (Figure 5a) [38]. The "swaposin" domain (d1qdma1) of aspartic proteinase prophytepsin has the same architecture, but the helices are in a different order (Figure 5b) [39]. Nevertheless, most of the structure comparison methods attempt to align the helices in agreement with their order along the sequence, which results in a visually poor superposition. They also fail to correctly align the cysteine residues forming the disulfide bonds. Only FlexSnap and DEDAL correctly handle these tasks (Figure 5c).

#### GTPases

**Figure 6:**
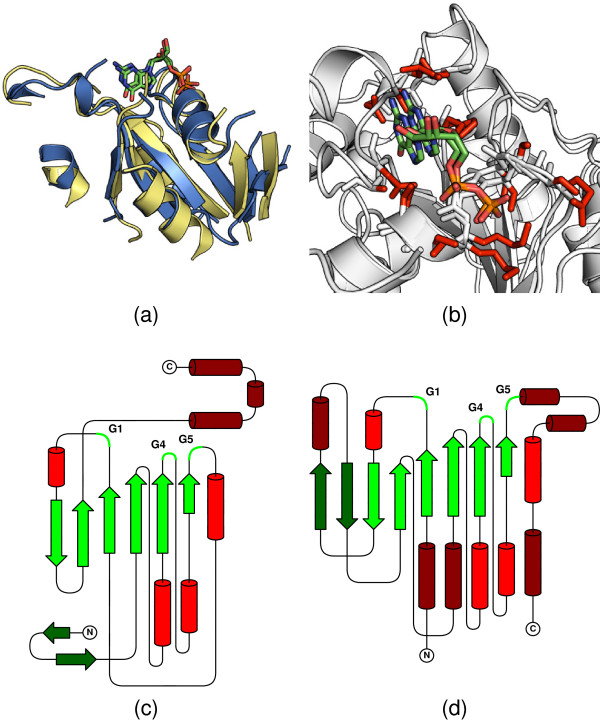
**Alignment of the Dynamin A GTPase domain and the cpGTPase domain from the YjeQ**. Topologies of (a) the Dynamin A GTPase (SCOP domain d1jwyb_) and (b) cpGTPase domain from the YjeQ protein (d1u0la2). Aligned SSEs are indicated by lighter colors. (c) DEDAL superposition of the GTPase and the cpGTPase domains (yellow and blue, respectively). For clarity, only the aligned parts of the structures are shown. (d) View of the binding site in the same superposition showing residues participating in the GDP/GTP binding (red) and the GDP molecule. Despite significant topological differences, DEDAL effectively handles all alignable SSEs and correctly superimposes the active sites. The sequence identity of the superimposed regions is 24.2%.

Guanine nucleotide-binding proteins (G proteins) control a range of cellular events. They act as *binary switches*, and use the GTP-GDP-GTP cycle to flip between the *on *and *off *states. They contain GTPase domains responsible for the GTP/GDP binding. It has been shown that the GTPase activity depends on the set of five conserved sequence motifs [40]. There exists an alternative circularly permuted GTPase structure (cpGTPase) [41] which contains all five motifs but in a different order (Figure 6a and 6b). Although having a different topology, the cpGTPase domains have the same architecture as GTPases, and retain the GTP binding activity. Despite the high sequence homology of the crucial motifs [42], many structure comparison methods are unable to correctly align residues which form the GTP/GDP binding site. CE and DALI yield 36% accuracy, while FlexSnap and C*_α_*-match have 90% accuracy (reference alignment contains residues responsible for GTP binding). In contrast, DEDAL yields an entirely accurate superposition in this region (Figure 6c and 6d).

#### Cyanovirin-N

**Figure 7:**
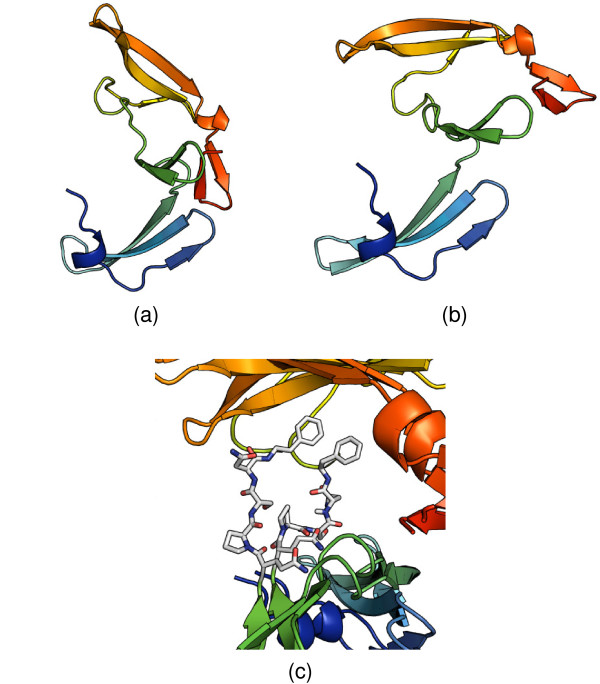
**Alignment of X-ray and NMR structures of the Cyanovirin-N**. Conformation of the Cyanovirin-N dimeric form depends on the molecular environment. (a) X-ray (d1l5ba_) and (b) NMR (d1l5ea_) structures have different conformations of the "hinge" region (PRO51-ASN53) (c). To fully analyze the similarity of the two structures it is necessary to abandon the rigid-body approach. The regions on both sides of the "hinge" have to be superimposed separately. DEDAL accomplishes this by extending local similarities in both regions and effectively defining the "hinge" as the boundary between them.

Cyanovirin-N is a potent HIV-inactivating protein, present in both monomeric and domain-swapped dimeric forms. Although the monomeric form is predominant in solution, and was determined first [43], the metastable dimeric form is also present. The dimeric form is stabilized in the crystalline state [44] and eventually its structure was also obtained by NMR [45]. For the dimeric form, it can be observed that the X-ray (SCOP domain d1l5ba_) and NMR (d1l5ea_) structures have a slightly different arrangement of subdomains (Figure 7a and 7b), and that the local conformations of all residues except for the hinge region (PRO51-ASN53, Figure 7c) are identical. Nevertheless, the similarity between the two structures cannot be easily determined by the rigid-body techniques, which align only one subdomain. Surprisingly FlexSnap, although in principle capable of handling conformational variability, gives only 50% accuracy with the reference alignment.

## Conclusions

DEDAL provides a direct approach to capturing similarity between proteins which is independent of rigid-body constraints. This is realized by systematically evaluating local structure context to identify similar regions of proteins while leaving aside regions which are different, where superposition is meaningless and should not be attempted. In addition, by focusing on local structure and carrying out a spatial rather than sequence attached analysis of matching substructures, it is not constrained by any particular order of structural features along the protein sequence. Because it identifies all local similarities between compared structures, it offers a rigorous and complete analysis. It is also very conservative in not extending the alignment beyond regions of pronounced structure similarity.

As structure comparison methods mature, the question as to whether compared structures are similar is being replaced by a need to determine the the exact nature of their similarity. The goal is to accurately indicate equivalent residues. Only manually curated alignments may be used to reliably assess this aspect of structure comparison. When tested on a relatively simple Conserved Domain Database [46], current automated techniques usually misalign residue pairs that are more than 3Å apart in the reference superposition, which amounts to 11 - 19% of the protein core residues [47]. This is also true for pairs of proteins within the same superfamilies, where even modest spatial divergence may lead to alignment errors [48]. On the more difficult test sets [7] (also used in this study), the quality of the alignments drops even further, to as low as 60% of the amino-acids correctly aligned over core and non-core residues. DEDAL represents a significant step forward in combating the above difficulties. While on the easier and medium difficulty test cases it is comparable to the best of other techniques, it outperforms them on the more demanding benchmarks. Thus, it effectively extends the ability to provide residue accuracy alignments to the most difficult cases, including discovering sequential permutations and spatial deformations. To our best knowledge, no other publicly accessible server offers this capability. The Linux binary of FlexSnap is publicly available but is less effective than DEDAL on both SISY and RIPC datasets. Local structure comparisons play an increasing role in the assignment of protein function [49,50]. DEDAL offers an effective technique for this class of applications. Furthermore, as recently demonstrated by Kosloff and Kolodny [51], assignment of function may also be helped by focusing on structural dissimilarity among proteins that are related by homology. By identifying only the significant local similarities, DEDAL allows effective differentiation between similar and dissimilar regions of structure, which could help guide functional assignments within protein families.

Because of the relatively large granularity of structure description and inclusion of the 3D structural context, DEDAL has the capacity for structure comparisons involving large sets of structures. Therefore it is well suited to improve automatic classification of structure domains, help analyze protein fold space, or to study molecular evolution processes. These areas reflect our future research interests. The presented methodology is being generalized to the structural multi-alignment problem.

## Methods

### Local Descriptors of Protein Structure

Descriptors have already been applied in several studies [27,28,52-55]. Here we use an improved version of the local descriptor methodology described in [28]. Every descriptor is built around its *central amino-acid*. In the first step, we identify residues close to the central amino-acid. For each pair of residues we compute distances between *C_α _*atoms (*d_α_*) and geometrical centers of side-chains *R_C _*(*d_C_*) (For glycine *R_C _*= *C_α_*, and for alanine *R_C _*= *C_β_*.). If either *d_α _*≤ 6.5Å, or *d_C_*≤ 8Å and *d_α _*- *d_C _*≥ 0.75Å (second condition favors residues whose side-chains point towards each other), we consider two residues to be in contact. In the second step, we build *elements *around selected residues by taking four sequential neighbours, two on each side. Finally, overlapping elements are merged into *segments*.

Thresholds used for contact determination are based on the range of intra-molecular interactions. However, in this study we came to the conclusion that contacts with distances close to their respective cutoffs require special treatment. Otherwise, when comparing two descriptors, an element which barely fits within a threshold in one descriptor might have a counterpart just outside of it in the second one. In such a case, two otherwise similar descriptors might be considered different. Therefore, we use a rough set approach [56]. We use a tightened set of thresholds for determining contacts (5.5Å and 7Å instead of 6.5Å and 8Å, respectively). If a contact satisfies lower thresholds, a corresponding element is considered *certain*. Otherwise, if it satisfies regular thresholds, it is considered *optional*.

Descriptors were designed to explore the structural neighborhood of their central amino-acid. Some descriptors, especially those built around surface residues, comprise only one or two segments. Frequently in this study, we refer to about three- or more segmented descriptors, which are expected to reflect the characteristics of a particular protein fold (e.g. three adjacent strands of a *β*-sheet). In the case of the hairpin-like motifs, segments are divided at the hairpin to mirror the secondary structure more accurately. This scheme of counting segments is required to properly define three-segmented descriptors as crucial to a given conformation and alignment, and was applied for the first time in this study.

To calculate the number of perceived segments of a descriptor, we first compute a spatial length of a segment by adding up distances between the averaged coordinates of three consecutive *C_α _*atoms. For example the length of a segment starting at the *m*^th ^and ending at the *n*^th ^residue *L_m..n _*equals:

$$L_{m..n}=\overset{n-1}{\underset{i=m}{\sum }}|{\bar{C}}_{\alpha }^{i}-{\bar{C}}_{\alpha }^{i+1}|$$

where ${\bar{C}}_{\alpha }^{i}=\frac{1}{3}\left(C_{\alpha }^{i-1}+C_{\alpha }^{i}+C_{\alpha }^{i+1}\right)$, and $C_{\alpha }^{i}$ are coordinates of the C*_α _*atom of the *i*^th ^residue.

Finally, we assume that segments longer than 18.0Å are in fact two "logical" segments connected by a short loop. The number of segments for a given length is computed as follows:

$$N_{m..n}=\left\lceil \frac{L_{m..n}}{18.0Å}\right\rceil$$

### Comparing descriptors

Fragment based methods typically use single segments of the same length, which are easy to compare, because the correspondence between residues (i.e. the alignment) is implicitly defined. In the case of descriptors, the alignment has to be computed as a part of the comparison process. If segments are of different lengths, all offsets have to be assessed. In the case of multisegment descriptors, all assignments of segments should, in principle, be tested (*k *segments imply *k*! alignments). The number of segments in descriptors may reach ten, giving over 10^6 ^potential alignments. Furthermore, it is unreasonable to demand that in similar descriptors all amino-acids should be aligned. To cope with these difficulties, we use a heuristic procedure based on the following principles:

1. central residues and their elements must be aligned exactly,

2. contacts between central residue and other residues must be preserved,

3. RMSD of aligned elements must not exceed 1.5Å,

4. for each pair of aligned elements, RMSD of substructures consisting of these elements and respective central elements must not exceed 2.5Å (i.e. elements should have the same position relative to the central element),

5. at least half of the segments must be aligned,

6. RMSD of aligned residues must not exceed 2.5Å.

We search through all alignments satisfying the conditions above. Firstly, we find all pairs of elements satisfying conditions 3 and 4. In the second stage, we construct all possible assemblies of those pairs and check for condition 6. If it is not met, these sets are reduced by removing the least fitting pairs of elements, until either condition 6 is met, or condition 5 is no longer satisfied. It should be noted that this process is totally sequence independent (i.e. the order of aligned segments in their respective proteins can differ). Because elements are the smallest indivisible blocks, it is possible that one segment will be aligned to two smaller ones which are a few residues apart. When computing condition 5, unaligned contacts which are optional in both descriptors are disregarded. It should also be noted that the approach of Bhattacharya et al. [18] uses a somewhat similar concept of local neighborhoods (*k *nearest residue neighbors) to carry out the structural alignment. They attempt to find a maximal common subgraph between their *k*-structures (in our case this task is accomplished through a contact guided systematic search). They report results for comparisons of 6 residues per neighborhood and note difficulties for comparing neighborhoods larger than 15 residues. Finally, they do not explore informational potential offered by the neighborhood approach to generate non-rigid body superpositions.

### Comparing structures

#### Graph representation and clique finding

In comparing two protein structures our first step is to find all similarities between their descriptors. All descriptors generated from the first structure are compared with all descriptors from the other, and alignments satisfying conditions described in the previous section are recorded. They are divided into two sets. The first set *S*_3 _contains alignments which have at least 3 segments. The second set *S*_1 _contains all the remaining alignments. The rationale behind this division is that alignments from *S*_3 _are likely large enough to encompass a significant similarity by themselves. Alignments in *S*_1 _are small and should be used only to extend structural alignments built with blocks from *S*_3_.

Each pair of aligned descriptors can be viewed as a partial alignment between structures. Such partial alignments can be combined to form a larger alignment if they are consistent in the overlapping parts or do not overlap at all. The solution computed by DEDAL is the largest (highest scoring) alignment that can be constructed from alignments of the individual descriptors. One should note that a set of partial alignments can be combined if and only if all its members are consistent with each other.

Finding the best alignment between structures is an extension of the clique finding problem in graphs. Let us assume that alignments between descriptors are nodes of an undirected graph *G*, and that there is an edge between two nodes if the corresponding alignments are consistent. In such case a clique in graph *G *can be interpreted as a valid alignment between the structures (Clique in a graph is a subset of nodes such that every two nodes in the subset are connected by an edge.). As long as the function used to score the alignments doesn't decrease with the clique growth, maximal alignments can be found by looking for the maximal cliques.

#### Accurate solution - TS and CTS algorithms

We use a branch-and-bound algorithm, which attempts to build all possible cliques, while preserving a required number of the highest scoring alignments. The algorithm traverses a decision tree, where each node corresponds to a decision whether to add a respective descriptor pair to the clique or not (nodes at the *k*^th ^level of the tree correspond to the decision of including the *k*^th ^graph node in the subset).

Obviously if a node cannot be a part of a clique in a given branch it is always rejected. In order to make this computation feasible we introduced two optimizations (cuts). A tree branch is abandoned if it is headed by a clique, which can be unambiguously expanded with a previously rejected node. In such a case all maximal cliques in that branch should contain that node, but such cliques belong to another branch of a decision tree. This ensures than only maximal cliques are obtained and each is constructed exactly once. Another optimization is based on the assumption that only the largest alignments (in terms of the number of aligned residues) should be considered. Therefore, if the lower bound of the size of a significant solution is already known (i.e. a sufficient number of alignments has already been found), it can be used to abandon certain tree branches as long as the estimate of the maximal alignment size is lower. Such estimate can be computed as a sum of a size of the alignment being built, and a number of residues outside this alignment covered by descriptor pairs, which are yet to be considered. Some of them are contradictory, and cannot be combined in one alignment, but still such upper bound is frequently low enough to abandon significant portions of a decision tree. We call this method a *Tree-Search algorithm *(TS).

We have also developed a modified version of the TS algorithm which extends the clique only if the subalignment which is being added has common residues with the alignment being extended. This mode can be used to make sure that the computed alignment comprises only one structurally continuous fragment. It is also used to extend alignments found by the TS algorithm in the set *S*_3 _with elements from *S*_1_. We call this algorithm a *Constrained Tree-Search algorithm *(CTS). In the second phase of the computation, either algorithm can be used to assemble elements from *S*_3_; CTS is always used during the third step. Abbreviations TS+CTS and CTS+CTS denote these two variants, respectively.

#### Monte-Carlo approximation

In certain instances, owing to the large number of nodes and edges in the graph, accurate algorithms are computationally infeasible. Such situations are most often caused by the size of the structures combined with a high degree of self-similarity (i.e. recurring structural motifs). Nevertheless, in these cases correct alignments are most likely easily identifiable by inspection. Therefore, it should be possible to easily detect them without a systematic search of the overwhelming solution space. Monte-Carlo methods [57] have a huge potential in finding low energy states of complex systems. We have implemented a Replica Exchange Monte Carlo algorithm to search for high score alignments. The REMC framework [58] is widely known and recognized. Here we will only describe the algorithm for generating transitions between states, and the energy function. Let *C_n _*= {*d*_1_, *d*_2_, ..., *d_n_*} be the clique defining a state at the *n*^th ^step. The clique *C_n_*_+1 _describing the state in the next step is generated as follows:

1. randomly pick a graph node *d *which doesn't belong to *C_n_*,

2. take a set *C_n_*_+1_containing *d *and elements from *C_n _*which are connected to *d *(one sees it is a clique),

3. if there are graph nodes which belong to every maximal clique containing *C_n_*_+1_, add them to *C_n_*_+1_.

The parameters of the REMC method (i.e. number of steps, number of replicas, their temperatures, and exchange frequency) have been chosen to reproduce accurate results in the shortest time. Our computational experiments have shown that in all tested cases REMC converges to the accurate solution.

#### Scoring function

Finding a useful alignment between two protein structures usually involves a compromise between the size of the alignment and its quality. Although DEDAL is designed to handle sequence permutations, segment swaps, etc., there are situations when it is desirable to construct alignments which preserve topology. Therefore, we introduce two control parameters: the maximal number of allowed sequence swaps (*M*), and maximal accepted sequence offset (*k*). If *M *is smaller than the actual number of swaps in the alignment, we compute only the largest sub-alignment containing at most *M *swaps. Sequence offset is used to obtain sequence dependent comparisons. It is assumed that there exists a direct 1:1 correspondence between the sequences of the proteins, and only residues aligned with offset not greater than *k *will be counted. This mode is especially useful for comparing models of the same protein in structure prediction applications [29,30]. Regarding the quality of the alignment, RMSD and other measures which evaluate distances between respective residues in a certain superposition are most useful if the alignment is constructed using a rigid-body strategy. In our case, every aligned residue pair belongs to at least one pair of similar descriptors satisfying the conditions given above. Thus the local alignment quality is already assured by similarity of respective descriptors. To evaluate the global quality we assess the spatial arrangement of the local components. We enumerate all pairs of the aligned residues which are in contact in at least one of the aligned structures. Then for each such contact we compute the RMSD of the respective five residue pieces (*elements*) of the backbone. These distances are averaged for each residue over all its contacts and for the whole alignment over all aligned residues. The result can be viewed as an average "tension" exerted on the two structures, when superimposed as elastic objects. This value raised to the power of 2 is subtracted from the number of aligned residues.

## Authors' contributions

PD implemented DEDAL, carried out computations and drafted the manuscript. BL provided substantial advice and guidance during all phases of the project. Both authors read and approved the final manuscript.

## Supplementary Material

Additional file 1 **Pruned SISYPHUS alignments - SCOP dataset**. The file contains SISYPHUS alignments chosen for the SCOP dataset. Click here for file

Additional file 2 **Pruned SISYPHUS alignments - MD dataset**. The file contains SISYPHUS alignments chosen for the MD dataset. Click here for file

Additional file 3 **Pruned SISYPHUS alignments - MC dataset**. The file contains SISYPHUS alignments chosen for the MC dataset. Click here for file

Additional file 4 **Figures S1 and S2**. Click here for file

Additional file 5 **Comparison of DALI and DEDAL performance on the SCOP subset**. Percentage scores of reconstructing the reference alignments on the SISYPHUS alignments SCOP subset. Click here for file

Additional file 6 **Comparison of DALI and DEDAL performance on the MD subset**. Percentage scores of reconstructing the reference alignments on the SISYPHUS alignments MD subset. Click here for file

Additional file 7 **Comparison of DALI and DEDAL performance on the MC subset**. Percentage scores of reconstructing the reference alignments on the SISYPHUS alignments MC subset. Click here for file

Additional file 8 **Comparison of DEDAL and other methods preformance on the SISY set**. Percentage scores of reconstructing the reference alignments on the SISY set. Results for other methods (except FlexSnap) cited after Mayr *et al*. [7]. Click here for file

Additional file 9 **Comparison of DEDAL and other methods preformance on the RIPC set**. Percentage scores of reconstructing the reference alignments on the RIPC set. Letters in the type column denote: R - repetitions, I - extensive indels, P - permutations, C - conformational changes. Results for other methods (except FlexSnap) cited after Mayr *et al*. [7]. Click here for file
