# Supplementary material for: A novel method to compare protein structures using local descriptors
Source: BMC Bioinformatics. 2011 Aug 17;12:344. doi: 10.1186/1471-2105-12-344 (PMC3179968; doi:10.1186/1471-2105-12-344)
Supplement: Additional file 4 — Figures S1 and S2. [file 1471-2105-12-344-S4.PDF]

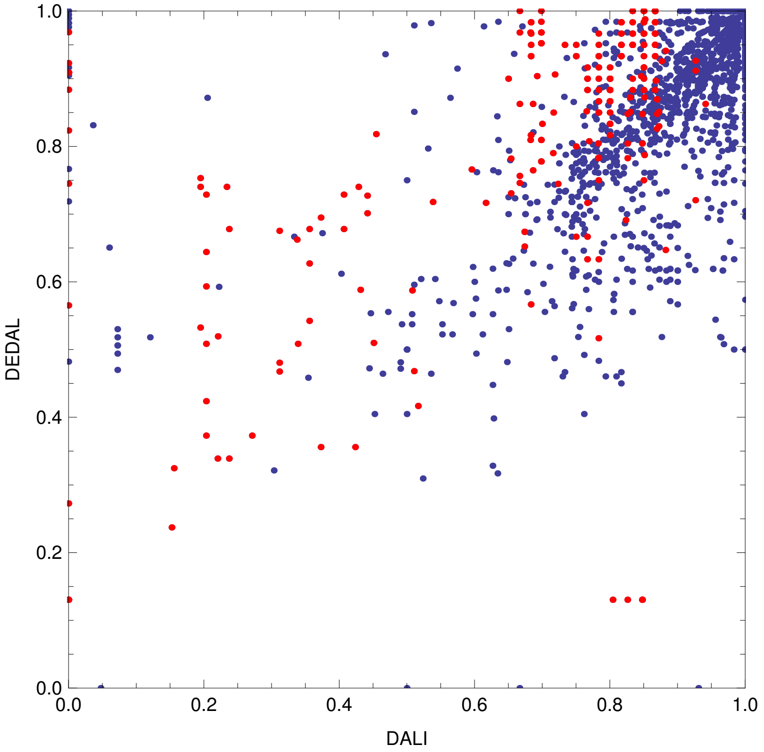

(a)

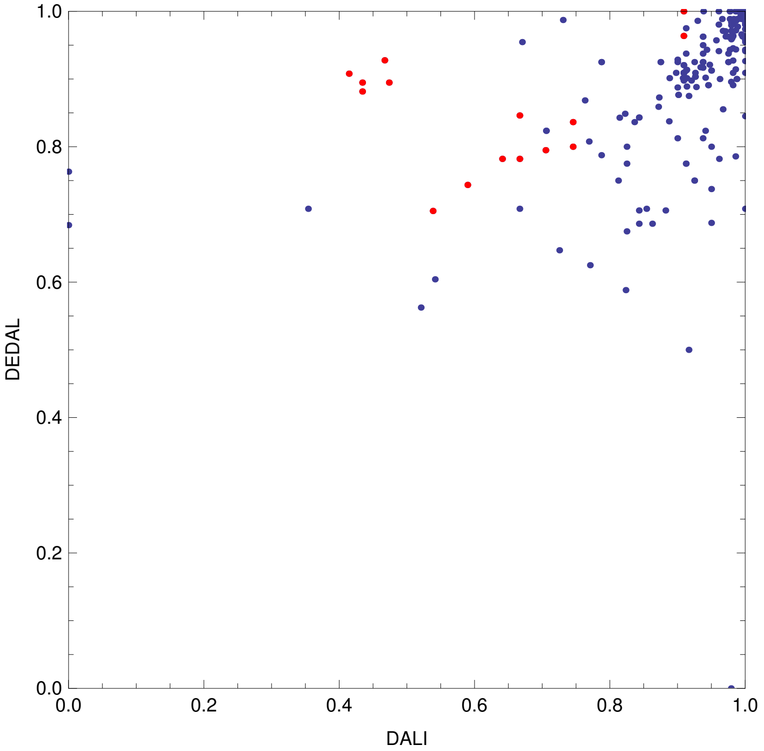

(b)

Figure S1: Percentage scores of reconstructing the SISYPHUS alignments from the SCOP (a) and MD (b) sets by the descriptor method and DALI. Red dots signify alignments with segment swaps or circular permutations.

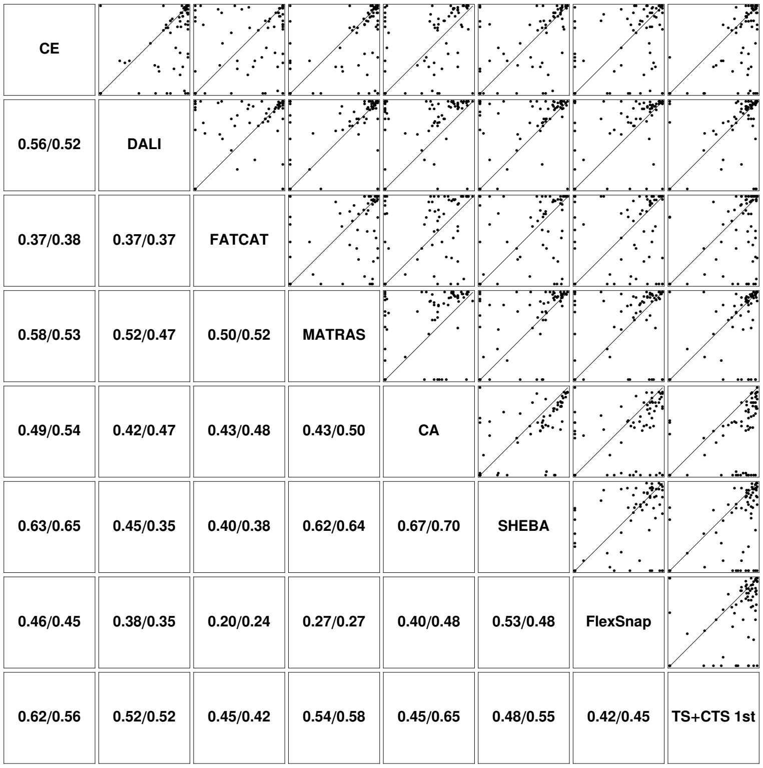

(a)

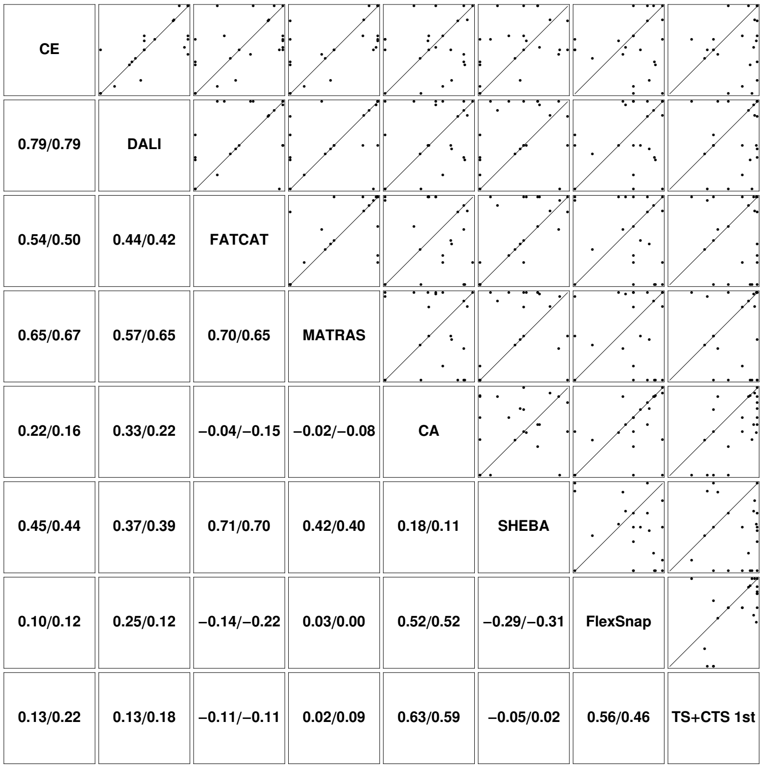

(b)

Figure S2: Correlation between the percentage scores of reconstructing the reference alignments by different alignment methods on the SISY (a) and RIPC (b) sets. Upper right triangle shows the scatter plots, while lower left shows the Pearson (first value) and Spearman (second value) correlation coefficients. Results for methods other than DEDAL and FlexSnap cited after Mayr *et al.*
